# Strengthening Modulus and Softening Strength of Nanoporous Gold in Multiaxial Tension: Insights from Molecular Dynamics

**DOI:** 10.3390/nano12244381

**Published:** 2022-12-08

**Authors:** Jiejie Li, Jie Li, Yangheng Chen, Jian Chen

**Affiliations:** College of Mechanical and Electrical Engineering, Central South University, Changsha 410083, China

**Keywords:** multiaxial loads, plastic deformation, molecular dynamics, nanoporous gold

## Abstract

The functionalized applications of nanoporous metals place clear requirements on their basic mechanical properties, yet there is a lack of research on the mechanical response under multiaxial loading conditions. In this work, the mechanical behaviors of nanoporous gold under multiaxial tension are investigated via molecular dynamics simulations. The mechanical properties under different loading conditions are compared and the microstructure evolution is analyzed to clarify the deformation mechanisms of nanoporous gold in biaxial and triaxial tension. It is found that the modulus of nanoporous gold in multiaxial tension is strengthened and the strength is softened compared to uniaxial tension. The failure of nanoporous gold in multiaxial tension is dominated by the progressive yielding, necking, and rupture of ligaments along the multiple uniaxial loading directions. The dislocation activity under multiaxial loads is more intense and more prone to plastic deformation, ultimately resulting in lower strength and smaller failure strain. The findings provide more insight into the understanding of the deformation mechanisms of nanoporous metals under complex stress states.

## 1. Introduction

Nanoporous metals (NPMs), which combine the structural optimization characteristics of porous materials and the superior properties of nanomaterials and metals, show excellent performances [1,2,3], such as ultrahigh specific surface area and strength, superior catalytic activity, and good tissue suitability, promising great potential for applications in chemical catalysis, shock absorption, sensing excitation, micro/nanodevices, and so on [4,5,6,7,8]. As a new kind of functional material, NPMs have been attracting a wide range of interest from researchers working to clarify the fundamental physical, mechanical, and chemical properties. Functionalized applications place corresponding demands on the mechanical properties of NPMs, and a better understanding of the correlation between microstructure and mechanical properties is essential for promising functionalization.

Since the proposal of “dealloying” [9] in 2004, a great deal of effort has been devoted to clarifying the mechanical behaviors of NPMs, and several theoretical, experimental, and numerical advances have been made. Nanoporous gold (NPG) has been used as a model material for studying the mechanical properties of NPMs because of its good stability and excellent properties. On the experimental side, the work focuses on characterizing the basic mechanical properties and deformation responses [3,10,11]. For example, Badwe et al. [10] conducted tensile experiments on NPG with various relative densities and ligament diameters, and the result showed that Young’s modulus obeyed a power law with relative density, but the exponent of 2.8 was higher than that predicted by Gibson–Ashby model. Furthermore, the fracture behavior of NPG showed a brittle-ductile transition with increasing ligament diameter. Theoretical work focuses on explaining the high strength and size-dependent properties of NPMs [12,13]. Research work in numerical computation focuses on uncovering the intrinsic mechanisms of specific deformation behaviors, as well as conducting large-scale simulations to develop corresponding scaling laws for the better prediction of mechanical properties. By simulating the spinodal evolution, Sun et al. [14] constructed an atomic model with stochastic bicontinuous structures efficiently characterizing the microstructure of the prepared NPG specimen by dealloying. The molecular dynamics results revealed the atomic mechanisms of dislocation multiplication, crack propagation, and structural failure. Li et al. [15] revealed the deformation mechanism of dislocation motion, grain boundary sliding, and grain rotation synergistically cooperating in nanocrystalline NPG. Roschning and Huber [16] derived the relationship between the structural disorder and mechanical properties of NPG under uniaxial compression using the finite element method, and the results showed an excellent agreement with experimental data, indicating that the scaling laws are efficient in predicting the macroscopic mechanical behavior of nanoporous materials.

The mechanical responses of NPMs in tension [10,17], compression [17], bending [17], and nanoindentation [18] have been widely explored, and these loading conditions are mainly under a single load or unidirectional loading. In actual service of functionalization, materials are inevitably subjected to the complex effects of multiple loads and these complex stress states will accelerate the damage and failure of materials. Due to the high demands placed on the equipment and specimens by the experiments, the mechanical behaviors of NPMs under complex loading conditions, such as multiaxial tension/compression, are rarely studied. Molecular dynamics (MD) simulations provide a useful and powerful tool to investigate the mechanical response of materials under complex loading conditions, and multiaxial tensile/compressive testing can simulate multiple planar stress states of materials. Hence, we explore the mechanical properties of NPG in biaxial and triaxial tension by using MD simulations. The differences in mechanical properties and deformation behavior under complex stress states are compared and discussed.

## 2. Methodology

**Figure 1:**
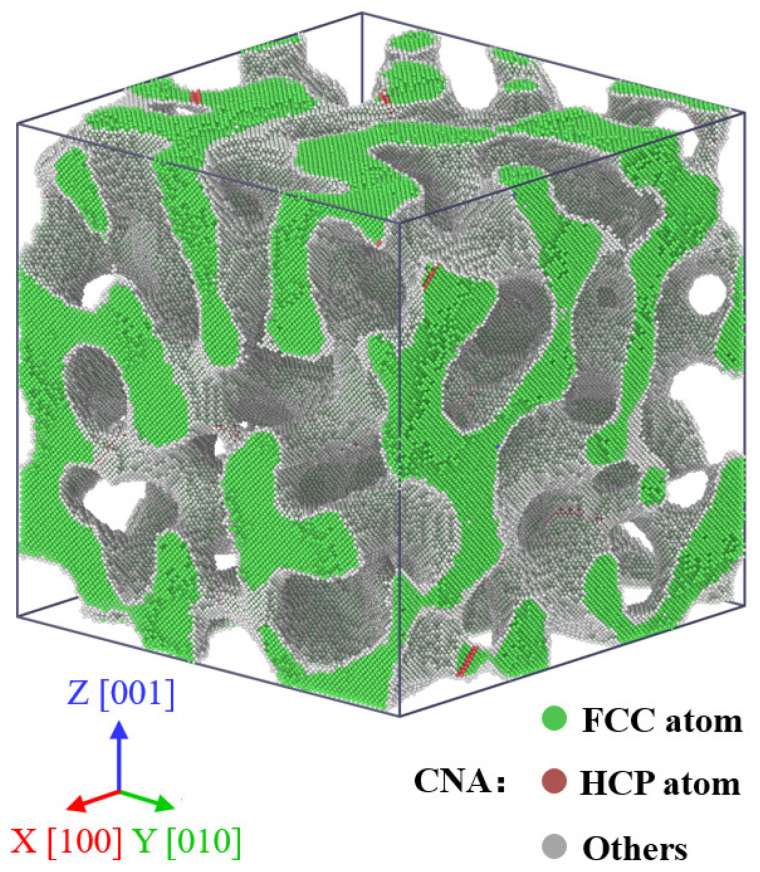
Atomic configuration of nanoporous gold with a relative density of ρ=0.40 and an average ligament diameter of d=4.7 nm. Atoms are colored by the CNA method, in which HCP atoms are colored red, FCC atoms are colored green, and other atoms are colored gray.

The previous study [19] has shown that the atomic configuration constructed by simulating the phase-field method is efficient in characterizing the microstructure of nanoporous materials. Herein, the spinodal decomposition is adopted to generate the nanoporous structure, which can be described by the Cahn–Hilliard equation [20]:

(1) $$\frac{\partial u}{\partial t}=\nabla^{2}\left[\frac{df(u)}{du}-\theta^{2}\nabla^{2}u\right]$$

in which u(x,y,z,t) is the difference in concentration of two simulated phases and lies in the range between −1 and 1, *t* is the evolution time, ∇ is the Laplace operator, f(u) is the free energy function, and θ is the width of the transition region between the two simulated phases. In this work, the double-well potential functions $f(u)={\left(u^{2}-1\right)}^{2}/4$ and θ=0.01 are utilized. The nanoporous structure is obtained by solving the Cahn–Hilliard equation numerically with the finite difference method in a cubic box spatially discretized into a 80×80×80 lattice. The details of the model solution are available in [14,19,21]. In this work, the initial cell dimensions of the NPG specimen are $80a_{0}\times 80a_{0}\times 80a_{0}$, where $a_{0}$ denotes the lattice of gold and $a_{0}=0.408\;\mathrm{nm}$ at 300 K. A NPG specimen with a relative density of 0.40 and an average ligament diameter of 4.7 nm is generated and investigated, as shown in Figure 1. The crystal orientations of the specimen along three orthogonal directions are X [100], Y [010], and Z [001], respectively. The atomic configuration is colored by the common neighbor analysis (CNA) method, in which face center cubic (FCC) atoms are colored green, hexagonal closed packing (HCP) atoms are colored red, and other atoms are colored gray.

All MD simulations are performed on the Large-scale Atomic/Molecular Massively Parallel Simulator (LAMMPS) [22]. Embedded atom method (EAM) potentials developed by Foiles et al. [23] are utilized to describe the interactions of gold atoms. Periodic boundary conditions are imposed on the three orthogonal directions of the simulation cell to mimic the bulk materials and eliminate the effect of specimen dimensions. For all simulations, the velocity-Verlet algorithm is used to perform numerical integration of motion with a constant time step of 1 fs. The initial specimen is relaxed to energy minimization using the conjugate gradient method, with maximum force and energy tolerances of $10^{-15}\;\mathrm{eV}\cdot {\mathrm{nm}}^{-2}$ and $10^{-15}$, respectively. After energy minimization, the simulation system is bathed at 300 K and 0 bar for 40 ps through an isothermal-isobaric (NPT) ensemble. Similar to previous studies [14,15,19], tensile loading is applied by the stepwise straining method. The loading is applied to the simulation cell through the fix/deform procedure with a fixed strain rate of $10^{9}\;s^{-1}$, and the specimen is remapped following the deformation of the simulated cell. In each loading step, the duration of loading is 1 ps, corresponding to an increment strain of ~0.001, and the system is relaxed for another 1 ps at 300 K. For uniaxial tension, the loading is applied in one specified direction. For multiaxial tension, the loadings are applied in multiple directions simultaneously. All visualizations are obtained in Open Visualization Tool (OVITO) version 3.7.10 [24], and the evolution of microstructure is analyzed by the CNA method. The engineering strain is defined as the elongation of the specimen dimension along the tensile tension. In multiaxial tension, the strain and stress of the specimen along different loading directions are calculated separately.

## 3. Results and Discussion

**Figure 2:**
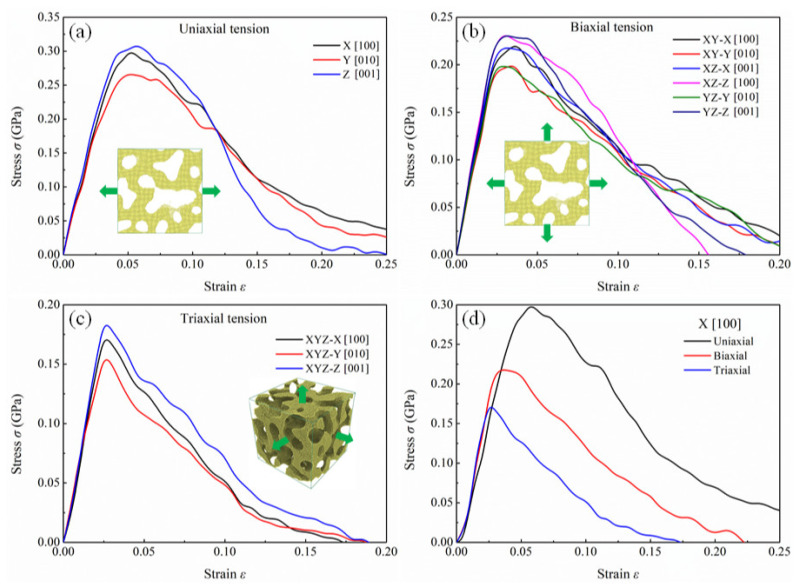
Engineering stress–strain curves of NPG in (**a**) uniaxial tension, (**b**) biaxial tension, and (**c**) triaxial tension. (**d**) Comparison of stress–strain curves of NPG along the X [100] direction under different loading conditions.

Figure 2a shows the engineering stress–strain curves of NPG in uniaxial tension along three orthogonal directions. The trends of curves along different directions are almost the same, including the elastic domain, hardening stage, and plastic deformation. However, there is a difference in stress between the three curves. It is observed that the ultimate stress along the X [100] direction is the highest, and the ultimate stress along the Y [010] direction is the lowest. The difference in strength and modulus reflects the anisotropy of the nanoporous structure. Theoretically, nanoporous structures constructed by solving the Cahn–Hilliard equation are homogeneous, as long as the dimensions of simulation cells are large enough. In real simulations, the simulation dimensions are finite to balance computational efficiency and resources. The simulation dimensions used in this work are limited to $32.64\times 32.64\times 32.64{\;\mathrm{nm}}^{3}$. Compared to the ligament diameter (about 4.7 nm), the box size is not sufficient to ensure that the microstructure of the NPG specimen is completely homogeneous, resulting in the above differences in mechanical properties of NPG along different loading directions. The engineering stress–strain curves of NPG in biaxial tension are depicted in Figure 2b. Under a load of biaxial tension, the stress–strain curves of the specimen along the fixed direction are coincident. Figure 2c shows the engineering stress–strain curves of NPG along three directions in triaxial tension. The curves are similar to those in uniaxial tension. For three different loading conditions, the ultimate stress and modulus of the specimen along the Z [001] direction are the highest, and the ultimate stress and modulus of the specimen along the Y [010] direction are the lowest, which is attributed to the inherent microstructure characteristics of the specimen. Figure 2d compares the stress–strain curves of the specimen along the X [100] direction for three loading conditions. Significant differences in the mechanical properties of specimens under different loading conditions are observed. At the same plastic strain, the material has the lowest stress in triaxial tension and the highest stress in uniaxial tension.

**Figure 3:**
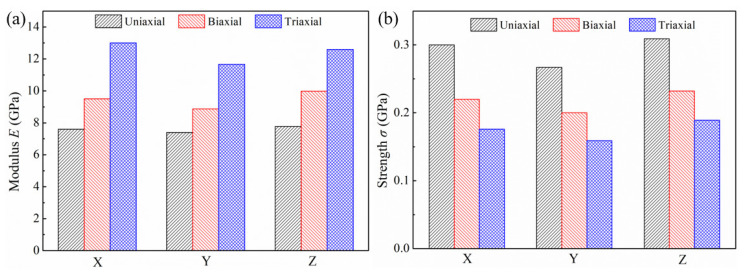
(**a**) Young’s modulus, and (**b**) ultimate strength of NPG under different loading conditions.

To quantitatively compare the mechanical properties of the specimen under different loading conditions, the ultimate strength and Young’s modulus are derived and illustrated in Figure 3a,b, respectively. The Young’s modulus is determined by the slope of the elastic domain regime of stress–strain curves. The average modulus and ultimate strength of the specimen in uniaxial tension are 7.59 GPa and 0.292 GPa. Using the scaling laws developed by Badwe et al. [10] and the Gibson–Ashby model [25], the predicted moduli of NPG with a relative density of 0.40 are 5.22 GPa and 12.64 GPa, respectively. Our simulated modulus is somewhere in between those values but closer to the experimental result, which verifies the reliability of the simulation results. The main reason why the simulated modulus is higher than the experimental result is that the experimentally prepared specimens have more defects, such as cracks, while the simulated model lacks the corresponding initial defects. At the same time, the high strain rate used in the MD simulations is also responsible for this discrepancy. In tensile experimental studies, the commonly applied strain rate is $1\times 10^{-3}\;s^{-1}$ [26]. However, due to the limitation of computing resources and efficiency, the strain rate used in the MD simulations usually ranges from $10^{8}–10^{9}\;s^{-1}$. The higher strain rate usually results in higher mechanical properties. Under the applied strain rate of $10^{9}\;s^{-1}$ in this work, the quantitative mechanical properties of the simulation may be higher. The overall trend of the curve is consistent with the experiment, indicating that the qualitative microstructure evolution behavior is feasible, which provides detailed insights with atomic-level spatial and temporal resolution to help us understand microscopic mechanisms behind deformation. Compared with uniaxial tension, the average modulus of the specimen in biaxial tension increases to 9.45 GPa with an increment of 24.5%, and the ultimate strength decreases to 0.217 GPa with a decrement of 25.7%. As for triaxial tension, the modulus (12.42 GPa) is enhanced by 63.6% and the strength (0.175 GPa) is softened by 40.1%. When the load changes from uniaxial tension to triaxial tension, the modulus of NPG increases, but the strength decreases. When subjected to multiple loads, the specimen is more likely to undergo plastic deformation, resulting in a reduction of strength. As indicated in Figure 2d, the failure strain also decreases with the loading changing from uniaxial tension to triaxial tension.

Furthermore, we analyze the deformation behaviors and microstructure evolution of NPG under different loading conditions. Referring to the previous study [14], the relative variations of HCP atoms and surface atoms are summarized and used to characterize the evolution of the microstructure. The variation of surface atoms is defined as $\xi =\left(N_{\varepsilon }-N_{0}\right)/N_{0}$, in which $N_{\varepsilon }$ and $N_{0}$ are the number of surface atoms at the applied strain of ε and at the initial state (ε=0), respectively. The increase in ξ is attributed to the generation of new surfaces. The fraction of HCP atoms is defined as $\eta =N_{\mathrm{HCP}}/N_{\mathrm{ALL}}$, in which $N_{\mathrm{HCP}}$ is the number of HCP atoms at different applied strains and $N_{\mathrm{ALL}}$ is the total number of atoms. The increase in η is mainly attributed to the formation of dislocations, including stacking faulting and twins [27].

**Figure 4:**
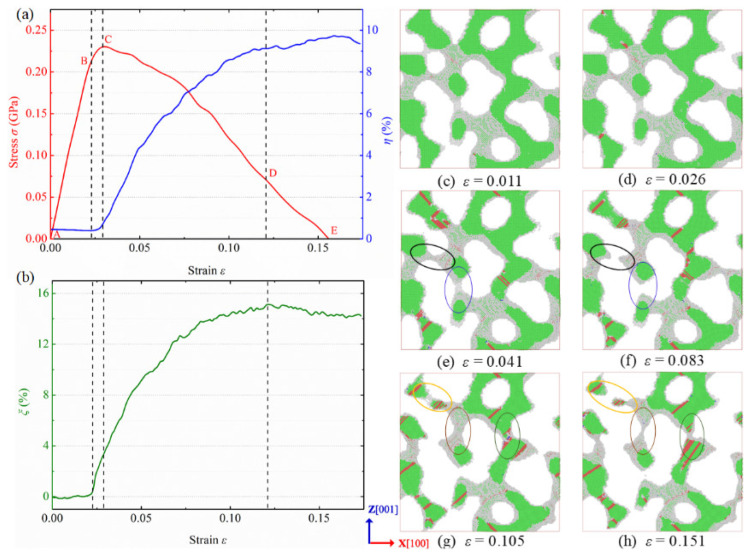
Variations of (**a**) stress along the X [100] direction (left, red), and HCP atom fraction η (left, blue), and (**b**) surface atom fraction ξ of nanoporous gold under the loading along X [100] and Z [001] directions with the applied strain. Microstructure evolution of NPG in biaxial tension with applied strains of (**c**), ε=0.011, (**d**) ε=0.026, (**e**) ε=0.041, (**f**) ε=0.083, (**g**) ε=0.105, and (**h**) ε=0.151. Atoms are colored by the CNA method, and a slice between y=19.3 nm and y=21.8 nm is shown.

The deformation behaviors and physical mechanism of NPG under uniaxial tension have been analyzed widely and are available in our previous study [19]. In this paper, we mainly focus on the deformation behaviors of NPG under multiaxial loading conditions. Figure 4a,b show the variations of stress, HCP atom fraction, and surface atom fraction of NPG in biaxial tension with the applied strain, respectively. The corresponding evolution of microstructure and dislocations are presented in Figure 4c–h. The whole deformation can be characterized by the following stage:

In the first stage (AB), the specimen undergoes elastic deformation, and the stress increases with the applied strain in linearity. The deformation is expected to be reversible after unloading, and the deformation of the ligament surface is negligible. There is no dislocation activity in the elastic deformation stage, as shown in Figure 4c. These two factors result in no evident increase in ξ and η.

After the yielding point (B), the deformation enters the second stage (BC). In this stage, the stress further increases with strain until the ultimate strength but in nonlinearity. The ligaments undergo plastic-yielding deformation, and new surfaces are generated, resulting in an increase in ξ. Moreover, the specimen begins to exhibit obvious dislocation activities, and partial dislocations and stacking faults are generated in the ligaments and the junctions of ligaments (Figure 4d), leading to a pronounced increase in η. Because the yielding deformation stage is short, the growth of HCP atoms is limited.

After the ultimate strength, the stress decreases with the applied strain. In the third stage (CD), the specimen undergoes large plastic deformation, and some ligaments exhibit necking, rupture, and even failure, as circled in Figure 4e,f. Both η and ξ show substantial growth, indicating a combination of intense dislocation activity, complex microstructural evolution, and large plastic rupture. At the applied strain of 12.1% (point D), most of the ligaments along the X [100] direction are fractured and most of the ligaments along the Z [001] direction are tightened, as indicated in Figure 4g.

In the last stage (DE), the specimen approaches its final breaking point, and the material fails, as depicted in Figure 4h. There is a presence of unintense dislocation activity, and the fraction of hcp atoms shows a slight increase. The fraction of surface atoms exhibits nearly the same and even a small decrease, illustrating a small change in surface area. Due to the difference in the microstructure of the specimen along the X [100] direction and Z [001] direction, the final breaking point might vary in different directions, which is also reflected by the failure strain in Figure 4b. For an individual ligament in NPG, the deformation and fracture mechanisms are similar to those of crystalline gold nanowires [14]. The deformation behavior of NPG in biaxial tension is similar to that in uniaxial tension, except for the necking and fracture of ligaments in multiple directions.

**Figure 5:**
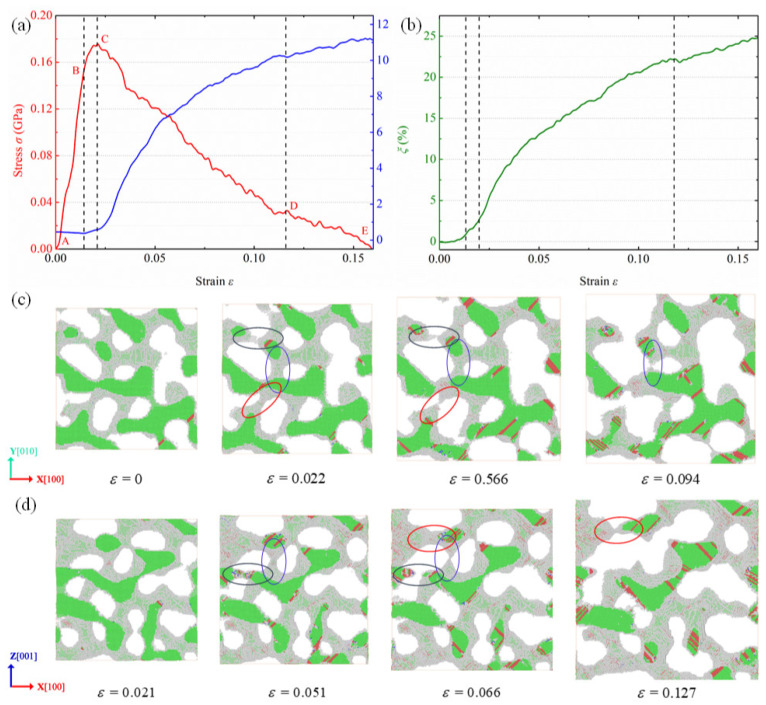
Variations of (**a**) stress along the X [100] direction (left, red), and HCP atom fraction η (left, blue), and (**b**) surface atom fraction ξ of NPG in triaxial tension with the applied strain. (**c**,**d**) Microstructure evolution of NPG in triaxial tension with different slices: (**c**) a slice between z=12.5 nm and z=17.5 nm, and (**d**) a slice between y=5.8 nm and y=10.8 nm.

Figure 5a,b show the variations of HCP atom fraction and surface atom fraction with the applied strain for the NPG specimen in triaxial tension. It is observed that the deformation behaviors of NPG in triaxial tension are similar to those in biaxial tension. Figure 5c,d show the microstructure and dislocation evolution of NPG in triaxial tension. The material fails by the yielding, necking, and rupture of ligaments. Due to the complex stress state, a small number of ligaments along the non-tensile direction are also fractured during the plastic deformation, as circled by the red markings in Figure 5c. As for the whole deformation, the failure of NPG in triaxial tension is dominated by the rupture of ligaments along the multiple uniaxial loading directions.

Further, we summarize the maximum increments of HCP atom fraction and surface atom fraction during the whole deformation. The maximum increments of surface atom fraction in biaxial tension and triaxial tension are 17.2% and 25.3%, respectively, which is about twice and three times as much as that in uniaxial tension (8.6%). The increase in surface atoms is mainly caused by the necking and fracture of ligaments in the NPG specimen, and the deformation of the specimen in uniaxial tension is dominated by the deformation of ligaments along the loading direction. In terms of the deformation degree alone, the deformation of the specimen under multiaxial loading conditions can be simplified as the superposition of deformation under uniaxial loads in multiple directions. The average increments of the HCP atom fraction of NPG in uniaxial tension, biaxial tension, and triaxial tension are 5.8%, 9.8%, and 11.5%, respectively. Under multiaxial loads, the specimen is subjected to greater and more complex loads, and the dislocation activity is more intense. The plastic deformation of the specimen is more intense, resulting in lower strength and smaller fracture strain.

## 4. Conclusions

The stress–strain responses and microstructure evolution of nanoporous gold in biaxial and triaxial tension are investigated, and the deformation mechanisms and mechanical properties are discussed. It is found that the modulus of NPG in multiaxial tension is strengthened and strength is softened compared to uniaxial tension. Under uniaxial load, NPG fails by the yielding, necking, and rupture of ligaments along the loading direction. Under multiaxial loads, the deformation of the specimen can be regarded as the superposition of the deformation of the specimen under multiple uniaxial loads. Under multiaxial loading, the specimen is subjected to greater and more complex loads, and the dislocation activities and plastic deformation are more intense, ultimately resulting in lower strength and smaller fracture strain. The findings further deepen the understanding of mechanical behavior and behind deformation mechanisms of nanoporous metals under complex stress states.

## Data Availability

The data that supports the findings of this study are available within the article.
